# 可视双腔支气管导管在胸外科手术中的应用和研究进展

**DOI:** 10.3779/j.issn.1009-3419.2022.101.35

**Published:** 2022-08-20

**Authors:** 诚 沈, 鹏 梁, 国卫 车

**Affiliations:** 1 610041 成都，四川大学华西医院胸外科 Department of Thoracic Surgery, West China Hospital, Sichuan University, Chengdu 610041, China; 2 610041 成都，四川大学华西医院麻醉科 Department of Anesthesiology, West China Hospital, Sichuan University, Chengdu 610041, China

**Keywords:** 可视双腔支气管导管, 胸腔镜手术, 单肺通气, 胸外科, Video double-lumen tube, Thoracoscopic surgery, One-lung ventilation, Thoracic surgery

## Abstract

以电视辅助胸腔镜技术为代表的微创胸外科，已逐步取代传统胸外科技术成为肺部结节包括早期肺癌等在内治疗的首要选择方式。随着双腔支气管导管在临床的应用，实现患侧肺隔离技术，不仅为胸外科微创手术的普及提供了坚实的麻醉基础，也为手术快速而平稳的实施提供了保障。然而，相较于单腔管而言，双腔支气管导管的管径更粗，管身较硬且难以塑形，给麻醉插管带来了不便；此外，由于双腔支气管导管的左、右支气管结构不同，麻醉插管过程中错位的发生率也很高。随着可视双腔支气管导管（video double-lumen tube, VDLT）在临床上逐渐使用，近年来成为胸科手术中的关注热点。本文就VDLT在胸外科手术中的应用和研究进展做一综述。

## Abstract (en)

The rapid development and promotion of minimally invasive thoracic surgery represented by video-assisted thoracoscopy surgery has gradually replaced traditional thoracic surgery technique as the primary choice for the treatment of pulmonary nodules, including early lung cancer. With the clinical application of double-lumen bronchial catheters, the realization of one-lung ventilation technology not only provides a solid anesthesia foundation for the popularization of minimally invasive thoracic surgery, but also provides a guarantee for the rapid and smooth implementation of the operation. However, compared with single-lumen bronchial catheters, the diameter of the double-lumen bronchial catheter is thicker, and the tube body is hard and difficult to shape, which brings inconvenience to anesthesia intubation. The bronchial structure is different, and the incidence of dislocation during anesthesia intubation is also high. With the gradual clinical use of video double-lumen tube (VDLT), it has become a hot spot in thoracic surgery in recent years. This article reviews the application and research progress of VDLT in thoracic surgery.

随着时间跨入21世纪，胸外科专业进入到一个飞速发展的阶段。微创外科、精准医疗、加速康复外科理念等在胸外科得到了广泛的体现和应用^[1-3]^。以电视辅助胸腔镜（video assisted thoracoscopic surgery, VATS）技术为代表的微创胸外科发展方向，已逐步取代传统胸外科技术，成为肺部结节包括早期肺癌等在内治疗的首要选择方式^[4-6]^。

与此同时，微创胸外科手术的成功与否更是与麻醉科医生的协同配合密不可分。麻醉医生行气管插管，保证手术侧肺塌陷良好的单肺通气（one-lung ventilation, OLV）技术，是胸外科手术尤其是肺部手术的必要条件^[7]^。气管导管第一次被明确记录用于临床治疗是在1885年由美国儿科医生Joseph O’Dwyer完成，当时他采用盲插技术置入金属导管拯救了一例白喉患者。此病例后，这种盲插技术在1888年的《纽约医学杂志》上第一次公开发表，并在同年被成功应用于胸科手术机械通气中。肺隔离技术早在1931年即被提出，并被首次将单肺通气运用于胸科手术。但问题是隔离侧肺中无法吸引分泌物且导管很容易移位。此后医学家们一直对该种导管进行改良却无法完全克服其先天缺陷。直到双腔支气管导管（double lumen tube, DLT）在临床的应用，才成功实现患侧肺隔离技术^[8]^，不仅为胸外科微创手术的普及提供了坚实的麻醉基础，也为手术快速而平稳的实施提供了保障^[9, 10]^。然而，相较于单腔管而言，DLT的管径更粗，管身较硬且在一定程度上难以塑形，给麻醉插管带来了不便；此外，由于DLT的左、右支气管结构不同，麻醉插管过程中DLT错位的发生率也很高^[11]^。麻醉插管中的定位也是另一个需要关注的问题。目前有多种方法辅助DLT定位，而纤维支气管镜（fiberoptic bronchoscopy, FOB）辅助定位被认为是DLT定位的金标准^[12]^。但是FOB的使用有一定难度，即使由有经验的麻醉医生操作也需要花费较长时间^[13]^。如何更加快速有效地建立单肺通气，是麻醉医生和胸外科医生都比较关心的问题。

随着可视双腔支气管导管（video double-lumen tube, VDLT）在临床上的应用，其已逐步成为研究的热点。它的外形与普通双腔支气管导管没有太大差异，最大的区别点就在于导管末端具有一颗高清摄像头，整个插管过程及手术中可以全程观察单肺通气的具体情况。本文就VDLT在胸外科手术中的应用及研究进展做一综述。

## 1 可视双腔支气管导管的组成

VDLT材质为无菌一次性塑料聚氯乙烯，具体包括了管体、微型摄像机、LED灯、视频器接口及冲洗系统^[14]^。与DLT相比，VDLT的导管末端是椭圆形，比DLT更粗，包括了两个发光二极管和一个微型传感器^[14]^。微型摄像机通过管壁内嵌的电缆与微型USB适配器及便携式外部高清显示器相连（图 1）。

**图 1:**
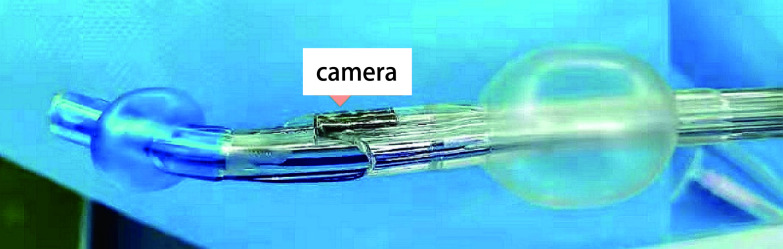
可视双腔支气管和前端的微型摄像头 Video double-lumen tube and the camera

外部连接的显示器可提供实时的高分辨率视频图像，以此可以清晰地看到导管位置及气道结构。此外，VDLT上的导管具有用于清除分泌物的冲洗通道，当VDLT前端的摄像头被密集分泌物如黏液或血液遮盖时，可通过冲洗系统用生理盐水进行清洁^[15]^。VDLT规格包括了左腔和右腔两大类，导管直径有Fr28、Fr32、Fr35、Fr37、Fr39和Fr41^[16]^。

## 2 临床常用的定位方法及可视双腔支气管导管的使用

常规的DLT气管插管有多种方法进行定位，包括了经典的听诊法、超声辅助定位法、呼气末二氧化碳分压监测法等，然而这些方法的定位成功率皆有限^[17]^。FOB是DLT定位的金标准^[18]^。与传统DLT定位不同，VDLT定位时可不用FOB辅助。此外，在FOB引导下进行DLT气管插管对麻醉医师技术要求高，操作过程一般都需要助手来帮助调整双腔管深度，人力资源要求高；而使用VDLT插管，对于麻醉医生来说更加容易操作及掌握，整个过程都可在屏幕监视下插入。一位稍作培训后的普通麻醉医师就能独立完成，顺利插管技术难度低^[19]^。

VDLT的具体操作过程如下：首先检查VDLT的气密性，试充盈导管套囊观察有无渗漏和破损。使用前用石蜡油将VDLT润滑，但应注意避免将润滑剂涂抹到镜头上。插管操作前，通过VDLT专用的连接线将电源数据线接头连接电源和监视器，调试后确保摄像头的图像能在监视器上显影再进行常规麻醉诱导。待患者肌松彻底起效后，利用可视喉镜直视下辅助插管。在VDLT插管过程中，将VDLT推进直到在外接显示器上看到患者清晰的隆突结构为止。若插管过程中监视器图像不清晰，可用2 mL生理盐水和20 mL空气反复冲洗相机镜头端口；若镜头依然模糊，则可使用FOB协助以确认导管的位置^[20, 21]^。在手术过程中，麻醉医生应连续在监视器屏幕上观察清晰显示的隆突结构以确认导管位置是否发生明显的移位。进行左VDLT插管时显示器清晰显示隆突及右侧支气管开口，在左主支气管中可以看到支气管袖带的上边缘；右VDLT进行插管时，显示器显示左侧支气管开口，并尽量使右侧导管侧开口对准右肺上叶开口^[21, 22]^。

## 3 可视双腔支气管导管在临床应用中的优势

### 3.1 插管时间

气管插管的时间定义为置入喉镜到套囊充气结束的完整时间。多项研究^[23, 24]^提示，VDLT的插管时间明显较传统DLT的时间要短。Onifade等^[12]^研究者通过纳入50例胸外科手术患者并将其随机行VDLT或普通DLT气管插管后发现，虽然两组插管的难易程度没有差异，但使用VDLT插管的时间明显更快（54 s *vs* 156 s, *P* < 0.001）。此外，VDLT组的插管错位发生率显著降低。Levy-Faber的研究成果^[25]^也得到了相似的结论，他们比较了VDLT组与传统DLT组的插管时间，发现使用VDLT可显著缩短插管时间和插管位置的视觉确认时间（51 s *vs* 264 s, *P* < 0.000, 1）。此外，在插管或手术期间，随机分配至VDLT组的患者均不需要进行FOB的检查。因此，与传统的DLT相比，VDLT能够显著加快插管速度，缩短插管时间。

### 3.2 插管成功率

Onifade等^[12]^研究发现，VDLT组的插管错位发生率相较于普通DLT组显著降低，所需的FOB辅助使用次数（28%）也显著减少。Rapchuk等^[26]^通过对72例患者采用VDLT的插管方式发现，95%的患者在第一次尝试时无需额外操作即可成功实现OLV的状态。仅有3例患者需要FOB的辅助。来自Heir等^[27]^研究者的实验结果也说明了类似的结论。这是一项随机对照实验，共纳入患者80例，患者在手术前被随机分配至DLT组或VDLT组。VDLT组通过其外部监视器而DLT组通过FOB提供的视图来验证插管的准确性。结果发现，VDLT组需要FOB来辅助纠正移位较普通DLT组明显降低（VDLT 7.7% *vs* DLT 100%, *P* < 0.000, 1）。

### 3.3 插管难易程度与学习曲线

首先，气管插管时对咽喉、气管黏膜的刺激会引起交感-肾上腺系统的活性增强，体内儿茶酚胺大量释放，患者在术中会出现例如心律增快、血压升高的临床表现，部分较严重者可出现心肌缺血、脑血管意外甚至心搏骤停。VDLT与普通的DLT相比，由于其具有插管、定位可视化的特点，降低了插管与定位的难度，避免了以往盲插与反复定位造成的不必要刺激^[28]^。其次，分析VDLT插管过程，利用末端处的摄像头清晰地观察到气道内部结构，并能及时调整导管前端的方向，使得双腔管与气管大致平行，更容易往下推送双腔管。整体操作过程降低了插管难度，缩短插管与定位的时间^[21, 29]^。此外，杜素贞等^[30]^研究者还通过绘制胸部微创手术中VDLT气管插管的学习曲线，深入了解麻醉医生学习VDLT气管插管的难易程度。研究者选择了6位能够熟练进行气管插管但之前从未使用过VDLT的住院医生。纳入90例择期行胸腔镜手术的患者，每位住院医师随机选择其中15例患者完成VDLT气管插管。结果发现，通过对6位住院医生学习过程的分析，成功完成VDLT气管插管的次数大约为9次，在此之后他们基本能够掌握操作技能。

### 3.4 全程可视

传统的DLT定位采用的听诊法和FOB辅助定位法。单纯依靠听诊，所得到的结果会因麻醉师的主观判断而受到影响。研究^[31]^发现，采用听诊法确定DLT放置满意后，经纤维支气管镜检查发现仍有37%的患者需要重新调整导管位置，而应用FOB辅助插管时导管错位率仍较高。与之相比，VDLT在气管插管过程中借助前端摄像头能全程清晰地看到气管隆突结构，更为直观化。

此外，由于全程可视，VDLT可以通过显示屏更加准确找到插管位置以减少患者口咽处受伤风险和缩短置管时间，为抢救赢得宝贵时间。熊振天等^[32]^报道了8例利用VDLT抢救窒息性大咯血的患者，最终取得了满意的效果。实际临床中，大咯血患者两侧肺存在大量淤血未能排出，使得普通DLT定位增加了难度，插管时间相应延长。传统听诊法定位也由于肺部的淤血残留，呼吸音不清晰，容易造成误听的结果。而对于FOB定位法，由于导管内壁附着血液，伸入导管内时，纤维支气管镜的前端容易被血液覆盖，影响视野。而VDLT弥补以上的弊端，利用良好的操作视野，麻醉医生可以在短时间内进行快速定位，有效确切地隔离双肺，保障通气安全。

### 3.5 降低气管插管操作后并发症

气管插管后最常见的并发症为咽痛（14%-90%）、声音嘶哑（10%-50%）和咳嗽^[22]^。多项研究^[25, 27]^发现，采用VDLT插管方式后，有效提高了患者满意度，降低了对患者气管和支气管的损伤的发生率，改善了患者咽喉部感觉。此外，针对这些并发症，有研究者^[33]^还提出利用右美托咪定联合VDLT的方式进行操作，主要考虑其是α2肾上腺素受体激动药，具有抗交感活性或拟迷走神经及镇静、镇痛作用。研究结果发现，与对照组相比，采用右美托咪定的研究组患者插管、拔管期平均动脉压、心率都比较平稳，患者插管过程中呛咳、躁动发生率低；术后气管及隆突黏膜损伤患者数量和程度都较普通插管组明显减轻；此外，右美托咪定联合VDLT组患者的术后咽痛、咳嗽等并发症的发生率也明显下降。

## 4 可视双腔支气管导管在临床应用中的劣势

### 4.1 发热

电子元件使用中最常见的问题就是由于长时间通电使用后带来的发热问题。VDLT在临床使用中有关发热的报道很少。既往有一篇报道^[34]^曾提到，由于VDLT摄像机故障产热导致导管前端软化变形。由于缺乏相关数据，VDLT长时间使用最大产热尚且未知，因此，麻醉医师在使用前应该仔细检查导管是否会发生过热，以免由于过度产热引发黏膜灼伤的相关危害。

### 4.2 运行稳定性

由于VDLT相较于传统DLT多了一些电子元件，在临床的使用运转过程中偶尔也会发生运行不稳定的情况。例如导管前端镜头与外部显示器的连接端口，有时会因为接触松动或接触不良导致显示器无法显像。在蓄电池蓄电不足的情况下，VDLT也会停止运行，给临床工作带来麻烦^[35]^。

### 4.3 外形的影响

由于VDLT前端带有摄像头，理论上其外径比同型号的普通DLT会更粗；Hoogenboom等^[21]^发现，由于VDLT外形与普通DLT有所不同，当部分需要FOB辅助下气管插管的情况变得更加困难。Dean等^[15]^提到，1例需FOB引导VDLT插管的患者，当FOB进入支气管导管时较容易，但其通过导管远端开口时遇到阻力，经润滑后才能顺利通过。

## 5 结论

随着VDLT的应用和开展，给临床工作带来诸多的便利。综合上述多项研究成果发现，使用VDLT进行麻醉气管插管时，可视化技术的应用提高了插管成功率，显著缩短了气管导管定位的时间，术中不仅可以持续观察隆突结构及时发现导管移位并调整，还能够在定位不良时快速重新定位而无需FOB的辅助^[36]^。VDLT支气管插管作为双腔管可视化技术的新进展，有必要进行更多的前瞻性随机对照临床研究，探讨其功能。同时，我们也要充分利用这些新技术增加临床诊疗的准确性和安全性，最大程度地降低医疗相关并发症，持续改进医疗质量。
